# Severe Imported Falciparum Malaria: A Cohort Study in 400 Critically Ill Adults

**DOI:** 10.1371/journal.pone.0013236

**Published:** 2010-10-08

**Authors:** Fabrice Bruneel, Florence Tubach, Philippe Corne, Bruno Megarbane, Jean-Paul Mira, Eric Peytel, Christophe Camus, Frederique Schortgen, Elie Azoulay, Yves Cohen, Hugues Georges, Agnes Meybeck, Herve Hyvernat, Jean-Louis Trouillet, Eric Frenoy, Laurent Nicolet, Carine Roy, Remy Durand, Jacques Le Bras, Michel Wolff, Severe Imported Malaria in Adults (SIMA) Study Group

**Affiliations:** 1 Service de Reanimation, Centre Hospitalier de Versailles, Le Chesnay, France; 2 AP-HP, Hopital Bichat-Claude Bernard, Departement d'Epidemiologie, Biostatistique et Recherche Clinique, Paris, France; 3 Institut National de la Sante et de la Recherche Medicale, CIE 801, Paris, France; 4 Service de Reanimation, Centre Hospitalier Universitaire Gui de Chauillac, Montpellier, France; 5 Service de Reanimation Medicale et Toxicologique, Centre Hospitalier Universitaire Lariboisière, Assistance Publique-Hopitaux de Paris, Paris, France; 6 Service de Reanimation, Centre Hospitalier Universitaire Cochin Saint Vincent de Paul, Assistance Publique-Hopitaux de Paris, Paris, France; 7 Service de Reanimation, Hopital d'Instruction des Armées Laveran, Marseille, France; 8 Service de Reanimation, Centre Hospitalier Universitaire Pontchaillou, Rennes, France; 9 Service de Reanimation, Centre Hospitalier Universitaire Henri Mondor, Assistance Publique-Hopitaux de Paris, Créteil, France; 10 Service de Reanimation, Centre Hospitalier Universitaire Saint Louis, Assistance Publique-Hopitaux de Paris, Paris, France; 11 Service de Reanimation, Centre Hospitalier Universitaire Avicenne, Assistance Publique-Hopitaux de Paris, Bobigny, France; 12 Service de Reanimation, Centre Hospitalier de Tourcoing, Tourcoing, France; 13 Service de Reanimation, Centre Hospitalier Universitaire Louis Mourier, Assistance Publique-Hopitaux de Paris, Colombes, France; 14 Service de Reanimation, Centre Hospitalier Universitaire de Nice, Nice, France; 15 Service de Reanimation, Centre Hospitalier Universitaire Pitié-Salpêtriere, Assistance Publique-Hopitaux de Paris, Paris, France; 16 Service de Reanimation, Centre Hospitalier Universitaire d'Angers, Angers, France; 17 Service de Reanimation, Centre Hospitalier Universitaire de Nantes, Nantes, France; 18 Service de Parasitologie, Centre Hospitalier Universitaire Avicenne, Assistance Publique-Hopitaux de Paris, Bobigny, France; 19 Service de Parasitologie, Centre Hospitalier Universitaire Bichat-Claude Bernard, Assistance Publique-Hopitaux de Paris, Paris, France; 20 Service de Reanimation, Centre Hospitalier Universitaire Bichat-Claude Bernard, Assistance Publique-Hopitaux de Paris, Paris, France; New York University School of Medicine, United States of America

## Abstract

**Background:**

Large studies on severe imported malaria in non-endemic industrialized countries are lacking. We sought to describe the clinical spectrum of severe imported malaria in French adults and to identify risk factors for mortality at admission to the intensive care unit.

**Methodology and Principal Findings:**

Retrospective review of severe *Plasmodium falciparum* malaria episodes according to the 2000 World Health Organization definition and requiring admission to the intensive care unit. Data were collected from medical charts using standardised case-report forms, in 45 French intensive care units in 2000–2006. Risk factors for in-hospital mortality were identified by univariate and multivariate analyses.

Data from 400 adults admitted to the intensive care unit were analysed, representing the largest series of severe imported malaria to date. Median age was 45 years; 60% of patients were white, 96% acquired the disease in sub-Saharan Africa, and 65% had not taken antimalarial chemoprophylaxis. Curative quinine treatment was used in 97% of patients. Intensive care unit mortality was 10.5% (42 deaths). By multivariate analysis, three variables at intensive care unit admission were independently associated with hospital death: older age (per 10-year increment, odds ratio [OR], 1.72; 95% confidence interval [95%CI], 1.28–2.32; *P* = 0.0004), Glasgow Coma Scale score (per 1-point decrease, OR, 1.32; 95%CI, 1.20–1.45; *P*<0.0001), and higher parasitemia (per 5% increment, OR, 1.41; 95%CI, 1.22–1.62; *P*<0.0001).

**Conclusions and Significance:**

In a large population of adults treated in a non-endemic industrialized country, severe malaria still carried a high mortality rate. Our data, including predictors of death, can probably be generalized to other non-endemic countries where high-quality healthcare is available.

## Introduction

*Plasmodium falciparum* malaria remains a major public health problem in endemic areas, with more than 1,000,000 deaths each year. About 12,000 cases of imported malaria are reported annually in Europe [1] and about 1300 in the United States [2]. Among non-endemic countries, France has the highest number of cases, about 5000 per year, of which 20 to 30 are fatal [3]; the second highest number is found in the United Kingdom, where the incidence is rising [4].

Severe imported malaria still carries a high mortality rate, which is estimated at 10% to 15%, although large studies are lacking [3]. One of the objectives of the World Health Organization (WHO) program for malaria control in Europe is to identify risk factors for death that can be used to optimize treatment decisions. In 2003, we reported several risk factors identified in 188 patients admitted to a single French intensive care unit (ICU) in 1988–1999 [5]. Although this study was the largest on managing severe malaria in a non-endemic industrialized country, it was conducted in a single center, over a long period, and used the 1990 WHO definition of severe malaria [6]. The WHO issued a new definition in 2000 [7]. Moreover, new treatment strategies for severe sepsis have been introduced over the last decade.

The objectives of this study were to gather multicenter data about severe imported malaria managed in the ICU in recent years, and to identify risk factors for mortality present at ICU admission.

## Methods

### Ethics Statement

Our study was approved by the Ethics Committee of the French Society for Critical Care Medicine (approval #07-211). Due to the retrospective design of the study, we did not obtain informed consent from the included patients. Nevertheless, all the data collected retrospectively were anonymized in a standardized case-report form and in the database.

### Study sites and population

The study was performed in the 45 adult ICUs in France that constitute the SIMA Study Group of 30 civilian university hospitals, three military teaching hospitals, and 12 regional nonteaching hospitals. Consecutive adults admitted to the 45 ICUs with a diagnosis of severe falciparum malaria between January 2000 and October 2006 were included and data were collected retrospectively (in 2006–2007) from the medical charts using standardized case-report forms.

### Definitions

According to the 2000 WHO definition [7], severe imported malaria was defined in this study as the combination of, (i) asexual *P. falciparum* forms in blood or a positive result of the *P. falciparum* histidine-rich protein 2 antigen-based rapid test, and (ii) one or more severity criteria (Table 1) at admission or within the first 2 days, and (iii) requirement of ICU admission (at the discretion of the ICU physicians). The 2000 WHO definition of severe malaria [7] was modified in part according to the SEAQUAMAT group [8]. More specifically, respiratory distress criteria were defined as stated in Table 1. Moreover, and to better characterize lung injury, we also defined respiratory failure as follows: in spontaneously breathing patients, PaO_2_ <60 mmHg on room air or need for supplemental oxygen and/or respiratory rate >32/min; and in patients receiving ventilatory assistance, PaO_2_/FiO_2_ ratio <200 mmHg.

**Table 1:** Clinical and biological criteria for severe malaria according to the 2000 World Health Organization definition with modifications (see * and †).

**Clinical criteria**
**Impaired consciousness**: Glasgow Coma Scale score <11*
Respiratory distress: requirement for noninvasive and/or endotracheal mechanical ventilation or spontaneous breathing with PaO_2_ <60 mm Hg (if FiO_2_ ≥0.21) †, and/or respiratory rate >32/min*
**Multiple convulsions**
**Circulatory collapse**: systolic blood pressure <80 mm Hg despite adequate volume repletion
**Abnormal bleeding**
**Jaundice**: clinical jaundice or bilirubin >50 µmol/L
**Macroscopic hemoglobinuria**: if unequivocally related to acute malaria (patients with blackwater fever were not included)
**Laboratory criteria**
**Severe anemia**: hemoglobin <5 g/dL
**Hypoglycemia**: blood glucose <2.2 mmol/L
**Acidemia** (pH<7.35) or acidosis (serum bicarbonate <15 mmol/L)
**Hyperlactatemia**: arterial lactate >5 mmol/L
**Hyperparasitemia** ≥4%
**Renal impairment**: serum creatinine >265 µmol/L or blood urea nitrogen >17 mmol/L*

*Coma scale criteria of 11 instead of 9; respiratory rate >32/minute and blood urea nitrogen > 17 mmol/L are modifications according to the SEAQUAMAT group [8].

† The requirement for noninvasive and/or endotracheal mechanical ventilation or spontaneous breathing with PaO_2_ <60 mm Hg (if FiO_2_ ≥0.21) was used specifically for this study.

Nonimmune patients were defined as Caucasians who traveled occasionally to endemic areas [5].

Community-acquired co-infection was defined as any infection diagnosed within the first 2 days after hospital admission. Infections occurring later were considered nosocomial. Acute lung injury (ALI) and acute respiratory distress syndrome (ARDS) were defined according to Bernard et al. [9].

### Management

Treatment, particularly the management of organ dysfunctions (including levels for blood products transfusions), was at the discretion of the ICU physicians. During the study period, recent and detailed French guidelines for the management of severe falciparum malaria were available [10]. These guidelines strongly recommended a quinine loading dose but did not recommend exchange transfusion.

Blood culture for microbiology and chest X-ray were performed routinely at ICU admission. Neuroimaging, electroencephalogram and hemodynamic investigations were performed if deemed necessary by the attending physician.

### Data collection

We recorded demographic data; previous medical history; country of malaria acquisition; chemoprophylaxis; clinical, laboratory, and imaging findings; and treatments and vital status at ICU and hospital discharge. Severity at ICU admission was assessed by computing the Glasgow Coma Scale (GCS) score [11], Simplified Acute Physiology Score II (SAPS II) [12], and Sequential Organ Failure Assessment (SOFA) score [13]. In each ICU, one intensivist completed a standardized case-report form for each patient. All case-report forms were reviewed by one of us (FB) for identification and resolution of inconsistencies.

### Statistical analysis

A descriptive analysis was performed on the overall sample. Categorical variables were described with numbers and percentages and continuous variables with mean and SD, or with median and interquartile range [IQR] for nonnormally distributed variables. Survivors and nonsurvivors were compared using the chi-square test for categorical variables and the Student's t-test or Wilcoxon rank-sum test, as appropriate, for continuous variables.

To identify risk factors for mortality, we constructed logistic regression models using a combination of multiple imputations and bootstrapping to select prognostic variables and to handle missing data [14]. Potential risk factors were as follows (*worst value during the 24 hours after ICU admission): sex, ethnic origin, co-morbidities, pulmonary failure*, shock*, age, time from symptom onset to ICU admission, GCS score*, plasma bicarbonate*, hemoglobin*, leukocytes*, platelets*, prothrombin time*, plasma creatinine*, highest blood glucose*, lowest blood glucose*, total serum bilirubin*, ALAT*, and parasitemia*. From the initial data set, we constructed 1000 bootstrap data sets and performed automatic stepwise logistic regression in each, selecting variables associated with mortality with *P*<20% by univariate analysis. Then, variables were selected for the second step if they were associated with mortality with *P* values <20% in at least 20% of the 1000 models. We implemented multiple imputation of missing data, which yielded five data sets. We then constructed 1000 bootstrap data sets for each imputed data set (i.e., 5000 data sets) and performed automatic stepwise logistic regression in each. Variables present in at least 60% of the 5000 models were selected for the final model. In the last step, we used the mean of the coefficient estimations obtained in each of the five imputed data sets. SAS 9.1 software (SAS Inc, Cary, NC) was used for all statistical analyses.

## Results

During the study period, 400 patients admitted to the ICU for severe falciparum malaria, were included. The median number of patients per center was 7 (IQR, 5–10; range, 2 to 35).

### General characteristics of the 400 patients

These characteristics are reported according to survival status in Table 2. Falciparum malaria was acquired in sub-Saharan Africa in 95.5% of the 366 patients for whom this information was available. Median stay duration in the endemic area was 1.0 month (IQR, 0.5–2.0). Of the 34.7% of patients who reported taking anti-malarial chemoprophylaxis, only 45.5% reported good adherence. Conditions associated with immune deficiency were noted in 7.3% of patients. One or more co-morbidities were present in 14.3% of patients. Five patients were pregnant.

**Table 2:** General characteristics of the 400 patients admitted to the intensive care unit with severe malaria by survival status.

Parameter	Survivors *n* = 358	Nonsurvivors *n* = 42	*P* value*
Mean age (±SD), years	42.8±15.0	55.6±14.2	<0.0001
Male, %	68.3	83.3	0.04
White, %	57.7	79.5	0.01
Black, %	36.0	12.8	
Other, %	6.3	7.7	
Previous malaria episodes, %	23.4	17.1	0.36
Antimalarial chemoprophylaxis, %	34.8	34.1	0.94
Nonimmune patients, %	43.8	59.5	0.05
At least one co-morbidity, %	13.1	23.8	0.06
Immune deficiency, %	7.8	2.6	0.34
Mean time from symptom onset to ICU admission (±SD), days	5.4±5.1	6.2±4.6	0.14

ICU, intensive care unit; SD, standard deviation.

*according to univariate analysis.

### Baseline patient characteristics and antimalarial therapy at ICU admission

The main clinical and laboratory characteristics and the pattern of WHO severity criteria during the first 24 hours in the ICU in survivors and nonsurvivors are reported in Tables 3 and 4, respectively.

**Table 3:** Baseline characteristics at intensive care unit admission in the 400 patients by survival status.

Parameter	Survivors *n* = 358	Nonsurvivors *n* = 42	*P* value*
SAPS II, mean±SD	30.2±15.6	70.6±26.3	<0.0001
SOFA score, mean±SD	7.7±3.8	15.4±5.2	<0.0001
Glasgow Coma Scale score, median [IQR]	15.0 [12.0–15.0]	9.0 [3.0–14.0]	<0.0001
Respiratory failure %	13.4%	45.2%	<0.0001
Highest temperature in °C, mean±SD	39.0±1.1	38.7±1.1	0.08
Arterial pH, mean±SD	7.41±0.1	7.19±0.2	<0.0001
Serum bicarbonates in mmol/L, mean±SD	21.7± 4.7	15.7±7.2	<0.0001
Arterial lactate in mmol/L, median [IQR]	2.4 [1.6–4.0]	8.4 [4.5–16.0]	<0.0001
Hemoglobin in g/dL, mean±SD	10.3±2.6	9.2±2.9	0.01
WBC·10^3^/mm^3^, median [IQR]	6.4 [4.7–9.6]	9.7 [5.9–17.9]	0.004
Platelet count·10^3^/mm^3^, median [IQR]	35.0 [19.0–57.0]	19.0 [13.0–29.0]	<0.0001
Plasma prothrombin time in %, mean±SD	72.6±16.8	53.4±26.8	<0.0001
Serum creatinine inµmol/L, median [IQR]	117.0 [87.0–228.0]	203.0 [156.0–302.0]	<0.0001
Lowest serum glucose in mmol/L, mean±SD	5.4±2.1	4.7±2.4	0.03
Highest serum glucose in mmol/L, mean±SD	9.6±4.5	12.3±5.9	0.0002
Total bilirubin in µmol/L, median [IQR]	51.5 [28.0–82.5]	99.0 [47.0–181.0]	<0.0001
Parasitemia first day in %, median [IQR]	6.0 [2.0–14.0]	17.0 [7.5–30.0]	<0.0001
Serum sodium in mmol/L, mean±SD	131.9±5.8	132.4±9.2	0.90
Serum potassium in mmol/L, mean±SD	3.8±0.6	4.1±1.1	0.09
Serum ALAT in IU, median [IQR]	58.0 [35.0–108.0]	104.5 [68.0–261.0]	<0.0001
Serum lactate dehydrogenase in IU, median [IQR]	966.0 [600.0– 1428.0]	2239.5 [1121.0–4128.0]	<0.0001
Serum C-reactive protein in mg/L, mean±SD	165.2±86.8	198.8±93.7	0.06

ICU, intensive care unit; SD, standard deviation; IQR, interquartile range; SAPS II, Simplified Acute Physiology Score II; SOFA, Sequential Organ Failure Assessment; WBC, white blood cell count; ALAT, alanine aminotransferase; IU, international units.

*according to univariate analysis.

**Table 4:** Severity criteria in the 400 patients at intensive care unit admission, according to outcome.

Severity criteria	Survivors *n* = 358	Nonsurvivors *n* = 42	*P* value*
Impaired consciousness, n (%)	77 (21.5)	26 (62.0)	<0.0001
Respiratory distress, n (%)	72 (20.1)	29 (69.1)	<0.0001
Multiple convulsions, n (%)	22 (6.2)	5 (11.9)	0.1849
Shock, n (%)	69 (19.3)	26 (61.9)	<0.0001
Abnormal bleeding, n (%)	7 (2.0)	3 (7.1)	0.0766
Jaundice, n (%)	178 (49.7)	30 (71.4)	0.0077
Hemoglobinuria, n (%)	20 (5.6)	2 (4.8)	1.0000
Severe anemia, n (%)	10 (2.8)	4 (9.5)	0.0453
Hypoglycemia, n (%)	5 (1.4)	8 (19.1)	<0.0001
Acidosis, n (%)	43 (12.0)	27 (64.3)	<0.0001
Hyperlactatemia, n (%)	37 (10.3)	26 (61.9)	<0.0001
Parasitemia > 4%, n (%)	223 (62.3)	34 (81.0)	0.0170
Renal impairment, n (%)	111 (31.0)	26 (61.9)	<0.0001
Mean number of severity criteria during the first 24 hours in the ICU (± SD)	2.4±1.6	5.9±2.4	<0.0001

WHO, World Health Organization; ICU, intensive care unit.

*according to univariate analysis.

Overall, median parasitemia at ICU admission was 7.0% (IQR, 2.7–15.0%). *P. falciparum* was identified in thin blood films in 84.7% of patients and/or in thick blood films in 50.7% and/or by antigen detection in 11.5%.

Intravenous quinine was used in 391 (97.8%) patients and other antimalarials in 9 patients. A quinine loading dose was used in 244 (61%) patients. The mean loading dose was 1072±359 mg, consistent with the mean body weight (72.7±14.5 kg) and French guidelines (quinine loading dose, 16 mg/kg) [9]. Of the 156 patients who received no loading dose, 9 did not receive quinine, 90 had received curative antimalarial treatment within hours before ICU admission, and 57 were not considered to require a loading dose (less severe form of malaria according to the physician in charge). An antibiotic was added in 36 patients. No patient underwent exchange transfusion.

### Complications of malaria and management

In 97 (24.3%) patients, at least one WHO defining criterion that was absent during the first 24 hours developed over the next 48 hours: impaired consciousness (n = 20), respiratory distress (n = 45), multiple convulsions (n = 6), shock (n = 26), abnormal bleeding (n = 6), jaundice (n = 12), hemoglobinuria (n = 2), severe anemia (n = 6), hypoglycemia (n = 5), acidosis (n = 10), hyperlactatemia (n = 7), hyperparasitemia (n = 4), and renal function impairment (n = 23).

Consciousness was impaired in 123 patients (103 at admission), for a mean duration of 2 days (IQR, 1–6; range, 1–67). Convulsions occurred in 33 patients, of whom 21 needed anticonvulsant therapy. Focal neurological abnormalities were present in 22 patients. Cerebral computed tomography (CT) and/or magnetic resonance imaging (MRI) were performed in 76 and 13 patients, respectively, and the findings were abnormal in 23 patients. At ICU discharge, 24 patients had at least one residual neurological abnormality: mental status abnormalities (n = 12), persistent focal deficits (n = 8), critical-illness polyneuropathy (n = 6), epilepsy (n = 2), and other abnormalities (n = 7).

On the first day, fluid resuscitation was given to 247 (61.7%) patients, including 213 (213/358, 59.5%) survivors and 34 (34/42, 81.0%) nonsurvivors (*P* = 0.0068). Throughout the ICU stay, 109 patients received catecholamines (norepinephrine, n = 81; and/or epinephrine, n = 26; and/or dopamine; n = 27; and/or dobutamine, n = 25), including 71 (71/358, 19.8%) survivors and 38 (38/42, 90.5%) nonsurvivors (*P*<0.0001).Low-dose steroids were used in 43 patients and activated protein C in only 3 patients.

At least one co-infection was diagnosed during the ICU stay in 96 patients (24.0%), including 78 (78/358, 21.8%) survivors and 18 (18/42, 42.9%) nonsurvivors (*P* = 0.0025). Community-acquired bacterial infections were diagnosed in 30 (30/96, 31.2%) patients including 25 (25/358, 7.0%) survivors and 5 (5/42, 11.9%) nonsurvivors (*P* = 0.2520). In the remaining 66 (66/96, 68.8%) patients with bacterial infections, the first episode was nosocomially acquired; 53 (53/358, 14.8%) of these patients were among the survivors and 13 (13/42, 31.0%) among the nonsurvivors (*P* = 0.0076). The main co-infection sites and microorganisms are detailed in the Table 5.

**Table 5:** Data on the 96 first episodes of co-infection in the 400 adults with severe imported malaria.

Parameter	Community-acquired infections n = 30	Nosocomial infections n = 66
**Pneumonia**Microorganisms(number of episodes)	**13 episodes** *Streptococcus pneumoniae* (3), MS *Staphylococcus aureus* (3), Gram+ *cocci* (negative culture) (1), *Escherichia coli* (1), *Haemophilus influenzae* (1), *Klebsiella pneumoniae* (1), *Enterobacter cloacae* (1), *Acinetobacter baumannii* (1), *Pseudomonas aeruginosa* (1), Not documented (2)	**48 episodes**MS *S. aureus* (11), *Streptococcus* sp (7), *S. pneumoniae* (3), MR *S. aureus* (1), *H. influenzae* (9), *E. coli* (4), *K. pneumoniae* (1), *Enterobacter aerogenes* (3), *E. cloacae* (2), *A. baumannii* (3), *P. aeruginosa* (5), *Bulkholderia cepacia* (1), *Legionella pneumophila* (1), *Citrobacter koseri* (1), Not documented (6)
**Bacteremia**Microorganisms(number of episodes)	**10 episodes** *E. coli* (3), *P. aeruginosa* (1), *Alcaligenes xylosoxidans* (1), *Clostridium* sp (1), *K. pneumoniae* (1), *Salmonella typhi* (1), *Campylobacter jejuni* (1), *Candida albicans* (1)	**8 episodes** *E. coli* (4), MS *S. aureus* (1), *Staphylococcus epidermidis* (1), *Klebsiella oxytoca* (1), *S. typhi* (1)
**Urinary tract infection**Microorganisms(number of episodes)	**3 episodes** *E. coli* (2), *Proteus* sp (1)	**9 episodes** *E. coli* (5), *K. pneumoniae* (3), *Enterococcus* sp (2)
**Other sites of infection**(number of episodes)	Abdominal infection (3), Skin and soft tissue (1)	Catheter infection (3), Sinusitis (2)

MS, methicillin-susceptible; MR, methicillin-resistant.

Endotracheal mechanical ventilation was required in 116 (116/395, 29.4%) patients, for a median duration of 6 days (IQR, 3–14; range, 1–201). In addition, 32 patients received noninvasive ventilation, which failed in 16 patients, who then received endotracheal mechanical ventilation. Thus, mechanical ventilation was used in 132 patients, of whom 90 survived and 42 died. Nonhemodynamic pulmonary edema was present in 76 (57.5%) of the 132 ventilated patients; among them, 58 had ARDS and 18 had ALI. The 56 other patients received ventilatory assistance essentially because of unarousable coma.

During the ICU stay, 81 patients required renal replacement therapy (intermittent hemodialysis, n = 64; and/or continuous veno-venous hemodiafiltration, n = 49).

Abnormal bleeding occurred in 23 patients. Blood transfusions were required in 114 patients, platelets in 63, and fresh plasma in 24. Overall, 845 blood-product units were transfused (538 blood, 177 platelets, and 130 fresh plasma units).

### Outcomes and factors predicting death

The overall hospital mortality rate was 10.5% (95% confidence interval [95%CI], 7.5%–13.5%), because all 42 deaths occurred in the ICU. The main characteristics of the nonsurvivors and causes of death are shown in Table S1. Death occurred within the first ICU week in 32 (76.2%) patients. Median ICU stay duration in the 42 patients who died was 3.5 days (IQR, 2–7; range, 1–198). Median ICU and hospital stay durations in survivors were 5 days (IQR, 3–8; range, 1–186) and 10 days (IQR, 7–17; range, 1–189), respectively.

By multivariable analysis, three variables present within 24 hours after ICU admission were significantly associated with death in the ICU: older age, lower Glasgow Coma Scale score, and higher parasitemia (Table 6).

**Table 6:** Independent predictors of death at intensive care unit admission in the 400 patients.

	N	OR (95%CI)	*P* value
Age (per 10-year increment)	400	1.72 [1.28–2.32]	0.0004
Glasgow Coma Scale score (per 1-point increment)	400	1.32 [1.20–1.45]	<0.0001
Parasitemia (per 5% increment)	400	1.41 [1.22–1.62]	<0.0001

R^2^ of the model: 0.90.

OR, odds ratio; 95%CI, 95% confidence interval.

## Discussion

In 400 adults with severe imported malaria, ICU mortality was 10.5%. Three variables present at ICU admission independently predicted death: older age, coma, and higher parasitemia.

To the best of our knowledge, this is the largest study of severe imported falciparum malaria in adults managed in a nonendemic country with high-level intensive-care facilities. It provides useful insights on disease outcomes and variables contributing to mortality, at least in France. These results should prove useful to clinicians managing severe malaria patients in the ICU, as well as to epidemiologists and public health practitioners studying potential risk factors for mortality in severe imported malaria. They may help to provide recommendations for intensivists, especially in countries where imported malaria is uncommon.

Whether our results can be generalized to other nonendemic countries deserves discussion. The few recent studies on severe imported malaria cannot be compared directly, for multiple reasons. Nevertheless, a number of points are shared by our study and recent studies from the UK and US [2], [4], [15], [16]: the majority of severe cases are related to *P. falciparum*, stay duration in endemic areas is around 20–30 days [4], [16], and fewer than 15% of patients take adequate chemoprophylaxis [2], [4], [15]. These common points suggest that our results may be at least partly relevant to other nonendemic countries having similar levels of intensive care. Finally, our results may serve as a reference for achievable survival rates and evaluations of complication rates in nonimmune individuals in resource-poor settings, where the investigations needed to characterize complications may be unavailable (e.g., routine high-quality chest X-ray, neuro-imaging studies, routine blood cultures, and hemodynamic measurements).

The definition of severe falciparum malaria issued by the WHO in 2000 is accompanied with data on the frequency and prognostic value of the defining criteria [7]. However, few such data are available for adults with severe imported malaria [3]. In our cohort, neither seizure activity nor hemoglobinuria was significantly associated with death in the univariate analysis. Among the other criteria, bleeding, anemia, and hypoglycemia are very uncommon in adults, and their prognostic relevance is therefore unclear. In our univariate analysis, six WHO criteria present within 24 hours of ICU admission were common and strongly correlated with ICU mortality, namely, impaired consciousness, respiratory distress, shock, metabolic acidosis, hyperlactatemia, and renal failure. Parasitemia >4% and jaundice were the two most common WHO criteria but were less strongly associated with mortality. We compared the main WHO severity criteria at admission in our 400 adults (including 182 nonimmune patients) and in 1050 Asian patients (including 11% young children and 26% older children) from low-transmission areas [17]. Globally, our population had (1) lower rates of coma, convulsions, acidosis, respiratory distress, and anemia; (2) similar rates of jaundice and renal failure; and (3) higher rates of shock and hyperparasitemia. Nevertheless, a large number of factors can influence the presentation, particularly access to healthcare (and more specifically to high-level intensive care) and time from symptom onset to hospital admission. Furthermore, the characteristics of the compared populations are often noticeably different in terms of age, proportion of pediatric patients, ethnic origin, level of anti-malaria immunity, background health status, and living standards.

Severe imported malaria continues to carry a high mortality rate even when high-quality healthcare is available and the affected population is chiefly composed of healthy adults. In our previous single-center retrospective study, mortality was 11% in the 93 patients with severe disease [5]. Among 76 episodes of severe adult imported malaria seen in the UK in 1991–2006, 8% were fatal [15]. These findings are consistent with our 10.5% mortality rate. In the two main trials of artemisinin derivatives versus quinine in adults with severe falciparum malaria in endemic areas, higher mortality rates of 15% *versus* 22%, and 13% *versus* 17%, respectively, were found [8], [18]. Although populations in endemic and nonendemic areas are not comparable, we believe the lower mortality in our study than in the quinine groups of these two trials is mainly ascribable to the availability of optimal treatments for organ failures and to the better baseline health status of our population.

Our multivariable analysis identified three factors present at ICU admission and predictive of death. Older age independently predicted death, in keeping with recent data from both endemic and nonendemic areas [17], [19]. This adverse impact of older age should receive careful attention given the increasing numbers of older travelers. Consciousness impairment assessed using the GCS score on the first ICU day also independently predicted death in our study. In our previous study, several factors, including impaired consciousness, were associated with death by univariate analysis, but the number of nonsurvivors was too small for a multivariable analysis [5]. Our findings in patients with severe imported malaria agree with the largest recent study conducted in endemic areas, which found that the main independent predictors of death were cerebral malaria (as assessed by the Glasgow Coma Score) and acidosis [20]. In contrast to studies from Africa and Asia, our study found no independent effect of acidosis at ICU admission on the risk of death. We included serum bicarbonate instead of arterial lactate in the multivariate analysis, because we had missing data on lactate levels, which probably contributed to the lack of significance of arterial lactate in the multivariate model. The missing lactate data can be ascribed chiefly to the retrospective study design, as arterial lactate was not measured routinely in patients at the lower end of the severity range, especially at the beginning of the study period. Unfortunately, base excess was not available on our case-report form. Nevertheless, bicarbonate and base excess, as measures of acidosis, were of roughly similar prognostic value, as shown in a recent study [20]. The third variable independently associated with death was parasitemia at ICU admission. The prognostic impact of parasitemia in severe malaria patients varies with multiple factors including geographic area, ethnicity, level of anti-malaria immunity, and previous exposure to antimalarial agents. Furthermore, parasitemia may chiefly reflect the circulating parasite load, which may not mirror the amount of parasites sequestered in the microvasculature. One group showed that total-body parasite biomass estimated from the plasma concentration of *P. falciparum* histidine-rich protein 2 (HRP2) predicted death more reliably than did parasitemia [21]. Few data are available on the prognostic significance of parasitemia in patients with severe imported malaria [3]. In our earlier study, median parasitemia was 18.2% in the 10 nonsurvivors and 3.5% in the 83 survivors (*P* = 0.02) but the difference was not significant after adjustment [5]. According to WHO guidelines, parasitemia >4% is sufficient to indicate treatment for severe malaria [22]. In a recent study including 482 cases of falciparum malaria, of which 72 were severe and 6 were fatal, the odds of having severe falciparum malaria were 12 times higher in patients with parasitemia ≥2% than in those with parasitemia <2% [15]. Further studies are needed to clarify the relevance of both parasitemia and plasma *P. falciparum* HRP2 as predictors of death in adults with severe imported malaria.

Although an earlier study showed that ethnic origin was an independent risk factor for severe malaria [15], ethnic origin did not independently predict death in our study. We cannot rule out a weak association of ethnic origin with mortality in patients who already have severe malaria, and our sample size may have been too small to detect a statistically significant effect.

Finally, to better assess the relevance of co-infections, we included “at least one infection” in the multivariable model. This parameter was not significant, suggesting that it did not independently predict death. In our setting, co-infections seem associated with severity rather than independently responsible for increased severity.

Our study has strong points compared to the main recent studies of severe imported malaria [5], [15]. The patient population is considerably larger and comes from 45 different centers, which increases the general validity of our findings. Furthermore, our study covers a recent period and therefore reflects the impact of the many recent advances in the management of severe sepsis [23], [24]. Finally, nearly all the patients received quinine therapy and, therefore, our results were not confounded by differences in the antimalarial drugs used. One limitation of our study is the retrospective design. However, the database was established by collecting information on consecutive patients using standardized data-collection forms. To obtain an adequate recruitment rate, we had to select adult ICUs experienced in the management of severe malaria, and consequently only 45 ICUs participated in the study. Some data were missing from the database. Nevertheless, this point was carefully taken into account and corrected by the statistical methodology using both multiple imputation and bootstrapping [14].

Our findings suggest several targets for improving the management of severe imported malaria. First, inappropriate chemoprophylaxis was common among both survivors and nonsurvivors. Efforts to improve adherence to chemoprophylaxis regimens are clearly needed. Second, we identified several independent predictors of death. A more standardized and aggressive treatment approach to patients with these predictors (and in particular immediately upon the onset of neurological impairment) might decrease the mortality rate. Third, we used quinine in nearly all our patients. A large study of adults with severe malaria conducted recently in Asia found that artesunate was associated with lower mortality rates and better tolerance, compared to quinine [8]. Thus, it can be hoped that the introduction of intravenous artesunate in nonendemic areas will improve survival rates in patients with severe imported malaria [25]. Unfortunately, intravenous artesunate is not yet available in France or other European countries. The only data on artesunate therapy for severe imported falciparum malaria come from a letter about 9 Norwegian patients, all of whom achieved a full recovery [26], and from the 2008 report of the Centers for Disease Control on 24 patients, of whom 1 died [2]. According to all these data, we wish to have parenteral artesunate, produced according to good manufacturing practices, in Europe.

In conclusion, severe imported falciparum malaria in adults still carried a high mortality rate in patients who received quinine and optimal supportive care in the ICU. The strongest predictors of death at ICU admission were older age, neurological impairment, and high parasitemia. Patients with these factors may require the highest level of treatment intensity. The results of our multicenter study are probably relevant to other nonendemic countries where high-quality healthcare is available.

## Supporting Information

Table S1 Nonsurvivors: Main characteristics and causes of death. Large table to describe the main characteristics and causes of death in the 42 nonsurvivors.
(0.10 MB DOC)
