# Correction: Severe Imported Falciparum Malaria: A Cohort Study in 400 Critically Ill Adults

**DOI:** 10.1371/annotation/e2552eb7-a70c-46f5-aca6-7526c56fb151

**Published:** 2010-10-26

**Authors:** Fabrice Bruneel, Florence Tubach, Philippe Corne, Bruno Megarbane, Jean-Paul Mira, Eric Peytel, Christophe Camus, Frederique Schortgen, Elie Azoulay, Yves Cohen, Hugues Georges, Agnes Meybeck, Herve Hyvernat, Jean-Louis Trouillet, Eric Frenoy, Laurent Nicolet, Carine Roy, Remy Durand, Jacques Le Bras, Michel Wolff, Severe Imported Malaria in Adults (SIMA) Study Group

The last name of the second author in the Hopital de Limoges collaborators in the SIMA study group was misspelled. The correct spelling is: D.Ajzenberg.

