# Supplementary material for: Severe Imported Falciparum Malaria: A Cohort Study in 400 Critically Ill Adults
Source: PLoS One. 2010 Oct 8;5(10):e13236. doi: 10.1371/journal.pone.0013236 (PMC2951913; doi:10.1371/journal.pone.0013236)
Supplement: Table S1 — Nonsurvivors: Main characteristics and causes of death. Large table to describe the main characteristics and causes of death in the 42 nonsurvivors. (0.10 MB DOC) [file pone.0013236.s001.doc]

**ONLINE SUPPORTING INFORMATION**

**Table S1. Nonsurvivors: main characteristics and causes of death**

| Patient | Age,  years | Number of  major WHO  criteria at  admission | Parasitemia  at admission  (%) | Time from ICU admission to death, days | Principal causes of death |
| --- | --- | --- | --- | --- | --- |
| Case 1 | 52 | 5 | 36 | 8 | Hypoglycemia on D5, coma, aspiration pneumonia, shock, ARDS, and possible myocarditis |
| Case 2 | 47 | 5 | 6 | 3 | MOF without evidence of bacterial co-infection |
| Case 3 | 57 | 9 | 80 | 2 | MOF without evidence of bacterial co-infection |
| Case 4 | 65 | 9 | 13 | 7 | MOF with pneumococcal pneumonia on D3 |
| Case 5 | 64 | 2 | 25 | 28 | ARDS complicating pneumococcal and *Haemophilus influenzae* pneumonia on D8. This patient had severe multiple sclerosis. |
| Case 6 | 38 | 6 | ND | 4 | Cardiac arrest 3 hours after ICU admission. *Escherichia coli* bacteremia (cholecystitis) |
| Case 7 | 50 | 3 | 30 | 6 | Shock and ARDS. No evidence of co-infection. |
| Case 8 | 58 | 7 | 30 | 3 | MOF without evidence of bacterial co-infection |
| Case 9 | 42 | 10 | 10 | 2 | MOF without evidence of bacterial co-infection |
| Case 10 | 52 | 4 | 0.1 | 42 | *Pseudomonas aeruginosa* pneumonia on D13 with ARDS |
| Case 11 | 44 | 6 | 5 | 1 | Seizures during a plane trip. Intubated at airport arrival. Multiple simultaneous organ failures with subsequent cardiac arrest during the quinine loading dose. |
| Case 12 | 52 | 5 | 7.5 | 6 | Bilateral pallidum necrosis on D3, massive cerebral edema, and cerebral herniation |
| Case 13 | 65 | 1 | 27 | 11 | Extensive myocardial necrosis on D3, shock and ARDS complicating bacteremic *E. coli* pneumonia |
| Case 14 | 92 | 8 | 35 | 2 | Shock and deep coma |
| Case 15 | 59 | 6 | 26 | 7 | Coma and *P. aeruginosa* pneumonia with shock and ARDS |
| Case 16 | 64 | 5 | 6 | 2 | Massive subarachnoid hemorrhage and massive cerebral edema, brain death |
| Case 17 | 62 | 3 | 50 | 5 | Shock and deep coma |
| Case 18 | 71 | 8 | 5.5 | 198 | MOF followed by multiple nosocomial infections. Refractory renal failure. |
| Case 19 | 39 | 5 | 20 | 2 | Massive cerebral edema, cerebral herniation, and brain death |
| Case 20 | 77 | 5 | 17 | 4 | Coma on D3, multiple organ failures, *P. aeruginosa* bacteremia |
| Case 21 | 67 | 8 | 15 | 3 | Massive cerebral edema.  This patient had Crohn’s disease. |
| Case 22 | 30 | 5 | ND | 7 | MOF complicating *Staphylococcus aureus* pneumonia |
| Case 23 | 46 | 6 | ND | 3 | MOF without evidence of co-infection |
| Case 24 | 48 | 4 | 15 | 13 | MOF. This patient had acute myeloblastic leukemia |
| Case 25 | 80 | 6 | 15 | 3 | MOF complicated by cardiac arrest.  This patient had diabetes mellitus. |
| Case 26 | 38 | 8 | 27 | 19 | MOF. Two episodes of nosocomial pneumonia, on D6 and D13. Secondary rupture of the spleen. |
| Case 27 | 74 | 3 | 12 | 2 | MOF without evidence of co-infection |
| Case 28 | 56 | 8 | 15 | 2 | MOF without evidence of co-infection. |
| Case 29 | 47 | 8 | 5 | 2 | MOF with *Klebsiella pneumoniae* pneumonia. |
| Case 30 | 54 | 6 | 30 | 3 | MOF with massive lactic acidosis complicated by intraventricular conduction block and subsequent cardiac arrest |
| Case 31 | 58 | 1 | 42 | 6 | Secondary MOF complicated by massive cerebral edema and subsequent brain death |
| Case 32 | 29 | 2 | 15 | 4 | MOF complicating fulminant bacteremic *E. coli* pneumonia |
| Case 33 | 42 | 9 | 25 | 1 | MOF and cardiac arrest |
| Case 34 | 71 | 7 | 0.1 | 11 | Coma; *P. aeruginosa* pneumonia on D2; multimicrobial nosocomial pneumonia on D6 complicated by ARDS (bilateral pneumothorax), shock, and subsequent MOF |
| Case 35 | 58 | 6 | 33 | 6 | Secondary coma and shock without evidence of co-infection followed by refractory MOF |
| Case 36 | 58 | 2 | 38 | 73 | Initial MOF. Four episodes of nosocomial infection (including late *Herpes simplex* virus pneumonia and late multisystem cytomegalovirus infection). Post-ARDS pulmonary fibrosis. Persistent renal failure and persistent coma. |
| Case 37 | 56 | 9 | 10 | 2 | MOF, with severe ARDS and shock |
| Case 38 | 25 | 7 | 50 | 3 | MOF with massive lactic acidosis complicating *E. coli* and *S. aureus* sepsis |
| Case 39 | 70 | 9 | 20 | 1 | MOF with rapid subsequent cardiac arrest. |
| Case 40 | 55 | 8 | 40 | 2 | MOF without evidence of co-infection. |
| Case 41 | 69 | 6 | 2.5 | 31 | *Enterobacter cloacae* pneumonia on D10. Persistent renal failure. |
| Case 42 | 57 | 6 | 3 | 2 | Severe *E. coli* community-acquired pneumonia on D1 complicated by subsequent MOF with severe ARDS and shock |

D, day; ARDS, acute respiratory distress syndrome; ND, not done; WHO, World Health Organization; ICU, intensive care unit; MOF, multiple organ failure
